# Proanthocyanidin Characterization and Bioactivity of Extracts from Different Parts of *Uncaria tomentosa* L. (Cat’s Claw)

**DOI:** 10.3390/antiox6010012

**Published:** 2017-02-04

**Authors:** Mirtha Navarro-Hoyos, Rosa Lebrón-Aguilar, Jesús E. Quintanilla-López, Carolina Cueva, David Hevia, Silvia Quesada, Gabriela Azofeifa, M. Victoria Moreno-Arribas, María Monagas, Begoña Bartolomé

**Affiliations:** 1 Department of Chemistry, University of Costa Rica (UCR), Sede Rodrigo Facio, San Pedro de Montes de Oca, San José 2060, Costa Rica; mnavarro@codeti.org; 2 Institute of Physical Chemistry “Rocasolano”, CSIC,C/ Serrano 119, Madrid 28006, Spain; rlebron@iqfr.csic.es (R.L.-A.); je.quintanilla@iqfr.csic.es (J.E.Q.-L.); 3 Institute of Food Science Research (CIAL), CSIC-UAM, C/ Nicolás Cabrera 9, Madrid 28049, Spain; carolina.cueva@csic.es (C.C.); victoria.moreno@csic.es (M.V.M.-A.); MJM@USP.org (M.M.); 4 IUOPA-Redox Biology Group, Department of Cellular Morphology and Biology, Faculty of Medicine, University of Oviedo, C/ Julian Claveria 6, Oviedo 33006, Spain; heviadavid@uniovi.es; 5 Department of Biochemistry, School of Medicine, University of Costa Rica (UCR), Sede Rodrigo Facio, San Pedro de Montes de Oca, San José 2060, Costa Rica; silvia.quesada@ucr.ac.cr (S.Q.); gabriela.azofeifacordero@ucr.ac.cr (G.A.)

**Keywords:** *U. tomentosa*, proanthocyanidins, propelargonidins, degree of polymerization, direct-injection mass spectrometry, antioxidant, antimicrobial, cytotoxicity

## Abstract

Apart from alkaloids, bioactive properties of *Uncaria tomentosa* L. have been attributed to its phenolic constituents. Although there are some reports concerning low-molecular-weight polyphenols in *U. tomentosa*, its polymeric phenolic composition has been scarcely studied. In this study, phenolic-rich extracts from leaves, stems, bark and wood (*n* = 14) of *Uncaria tomentosa* plants from several regions of Costa Rica were obtained and analysed in respect to their proanthocyanidin profile determined by a quadrupole-time-of-flight analyser (ESI-QTOF MS). Main structural characteristics found for *U. tomentosa* proanthocyanidins were: (a) monomer composition, including pure procyanidins (only composed of (epi)catechin units) and propelargonidins (only composed of (epi)afzelechin units) as well as mixed proanthocyanidins; and (b) degree of polymerization, from 3 up to 11 units. In addition, *U. tomentosa* phenolic extracts were found to exhibit reasonable antioxidant capacity (ORAC (Oxygen Radical Absorbance Capacity) values between 1.5 and 18.8 mmol TE/g) and antimicrobial activity against potential respiratory pathogens (minimum IC_50_ of 133 µg/mL). There were also found to be particularly cytotoxic to gastric adenocarcinoma AGS and colon adenocarcinoma SW620 cell lines. The results state the particularities of *U. tomentosa* proanthocyanidins and suggest the potential value of these extracts with prospective use as functional ingredients.

## 1. Introduction

Proanthocyanidins are condensed flavan-3-ols with a high degree of structure variability, depending on their constituent monomers structure (propelargonidins, procyanidins, prodelphinidins, profisetinidins and prorobinetinidins), their interflavanic bond type (type A and type B proanthocyanidins), and its degree of polimerization (DP). Proanthocyanidins have become of high interest because of their biological properties, such as antioxidant, anti-inflammatory and anticancerigen properties with further investigation of interest due to proanthocyanidins potential use in cancer prevention [1].

*Uncaria tomentosa* L., also known as cat’s claw, is a plant used in traditional medicine, distributed in South America, mainly in Peru and Brazil as well as in Central America. There are numerous scientific reports on *U. tomentosa* bioactivity, comprising anti-inflammatory and antioxidant properties, and protective effects against cancer, as well as positive effects in the cardiovascular, central nervous and locomotor systems [2]. These bioactive properties of *U. tomentosa* have been attributed mainly to its alkaloid contents [3], however it has been reported that some properties such as its antioxidant effect could be related to its phenolic contents [4].

On the other hand, the antimicrobial activity of *U. tomentosa* has received little attention [5,6]. Phenolic compounds are recognized as antimicrobial agents on certain human bacteria. Some of them, such as hydroxybenzoic and hydroxycinnamic acids have shown antimicrobial effects against *Salmonella* [7] while monomeric flavonols are also capable to inhibit the growth of pathogen bacteria such as *Clostridium perfringens*, *C. dificile*, *Bacteriodes* spp., while probiotic bacteria strains from *Lactobacillus* and *Bifidobacterium* seems to be less sensitive [8]. In the case of proanthocyanidins, it has been described that a prolonged administration of these compounds produced a change in microbial population towards Gram-negative species (i.e., *Enterobacteriaceae* and *Bacteriodes*), indicating a greater susceptibility of Gram-positive bacteria to be inhibited by high molecular weight polyphenols [9].

*U. tomentosa*’s recent work on low-molecular weight polyphenols using liquid chromatography coupled to a triple quadrupole mass spectrometer indicated a high flavan-3-ol content, including procyanidin dimers and trimers, propelargonidin dimers, and cinchonain-type flavalignans (epicatechins substituted with phenylpropanoids), some of which were reported for the first time in *U. tomentosa* [10]. However, the study of greater molecular weight polyphenols requires the use of mass spectrometers with a broader *m/z* range, such as those based on Time of Flight (TOF) analysers. Thus, the objective of the present work was to perform a detailed characterization study of polymeric proanthocyanidins of *U. tomentosa* extracts using direct-injection (DI) mass spectrometry on an instrument equipped with electrospray ionization and a quadrupole-time of flight analyser (ESI-QTOF MS). In addition, extracts were assayed for different bioactivities, in particular, antioxidant activity, antimicrobial activity against respiratory pathogens and antitumoral effects in gastric and colon cancer cell lines. Finally, possible relationships between these activities and total proanthocyanidin content of the *U. tomentosa* extracts were explored.

## 2. Materials and Methods

### 2.1. Plant Material, Chemicals and Reagents

*Uncaria tomentosa* samples were collected from different places in Costa Rica: Asomat (northern part) and Aprolece-Palacios (Caribbean part), and others growing in the wild in Los Chiles (northern part) and Sarapiqui (Caribbean part). Vouchers for all plants are deposited in the Costa Rican National Herbarium, under series no. AQ2953, AQ3331, AQ3332 and AQ3510, respectively. The plant material was separated in its different parts: leaves (H), stems (T), bark (C) and wood (M), and then dried in a stove at 40 °C, being turned over every 24 h for a week until totally dry. The dried material was then ground and preserved in plastic recipients.

MTBE (methyl tert-butyl ether), chloroform, hexane, and methanol were purchased from Baker (Center Valley, PA, USA). Minimum essential Eagle’s medium (MEM) containing 10% fetal bovine serum (FBS), glutamine, penicillin-streptomycin, and amphotericin B were obtained from Life Technologies (Carlsbad, CA, USA). Trypsin-EDTA solution, DMSO (dimethyl sulfoxide) and MTT (3-(4,5-dimethylthiazol-2-yl)-2,5-diphenyltetrazolium bromide) were provided by Sigma-Aldrich (St. Louis, MO, USA).

### 2.2. Extraction of Phenolic Compounds from the Different Parts of U. tomentosa

Phenolic extracts from U. tomentosa leaves, stems, bark and wood were obtained as previously described [10]. Briefly, non-polar compounds were first extracted from the dried material in a mixture (0.05 mg/mL) of methyl ter-butyl ether (MTBE) and methanol (MeOH) 90:10 (*v*/*v*) at 25 °C during 30 min in ultrasound folllowed by letting the mixture standing for 24 h, and, after filtration, repeating the extraction process once. The solvent was evaporated to dryness and the residue washed with MeOH to extract residual polyphenols. After the MTBE-MeOH extraction, the polyphenolic-rich extract was obtained by extracting the residual plant material with MeOH at 25 °C during 30 min in ultrasound followed by letting the mixture standing for 24 h, and, after filtration, repeating the extraction process twice. These methanol extracts and the MeOH washings were combined and evaporated to dryness, under 40 °C, followed by succesive washings with hexane, MTBE and chloroform.

### 2.3. Total Proanthocyanidin Determination

Total proanthocyanidin content was determined by a modification of the Bate-Smith method, which is based on the acid-catalyzed oxidative cleavage of the C–C interflavanic bond of proanthocyanidins in butanol-HCl [11]. Briefly, 0.2 mL of each extract and 20 mL of butanol/HCl (50:50) (0.54 mM FeSO_4_) were incubated at 90 °C for 1 h. After cooling, the mixture volume was made up to 25 mL with butanol-HCl mixture, and the absorbance was measured at 550 nm against a blank prepared in the same way but without heating. Cyanidin chloride was used as standard to construct the calibration curve. Results were expresses as mg of cyanidin chloride equivalents/g of extract.

### 2.4. Characterization of the Proanthocyanidin Extracts by DI-ESI-QTOF MS

Mass Spectrometry experiments were carried out with an Agilent 1200 Series Liquid Chromatography system (equipped with a binary pump, an autosampler and an column oven) coupled to a 6520 quadrupole time-of-flight mass spectrometer using an electrospray interface (DI-ESI-QTOF MS instrument hereafter). All instruments were from Agilent Technologies (Santa Clara, CA, USA). 5 mg of the phenolic extracts were dissolved in 1 mL of methanol:water (1:1, *v*/*v*) and the solution filtered through 0.45 µm. 20 µL of the filtrate were injected using the autosampler into the LC system (without column) and carried through in acetonitrile:water (3:1, *v*/*v*) eluent at a flow rate of 100 µL/min [12]. The ESI source parameters were previously optimised, and adjusted as follows: spray voltage 4.5 kV; skimmer voltage, 300 V; fragmentor voltage, 500 V; drying gas temperature 300 °C; drying gas flow rate 6 L/min and nebulizer pressure 30 psi. Nitrogen (99.5% purity) was used as drying and nebulizer gas. Mass spectra were acquired in the negative mode, recording from *m/z* 100 to 5000. Data acquisition and processing were done using Agilent Mass Hunter Workstation Acquisition v. B.02.00 software (Agilent Technologies, Santa Clara, CA, USA). Assignation of DI ESI-QTOF MS signals (*m/z*) to a particular proanthocyanidin structure (i.e., propelargonidins, procyanidins, and prodelphinidins) was achieved by the calculation of the theoretical monoisotopic mass (as deprotonated ion, [M−H]^−^), according to the Equation:

[M−H]^−^ (*m/z*) = 290.0790 × (EPI)CAT + 274.0841 × (EPI)AFZ − 2.0156 × (B) − 1.0078

where (EPI)CAT and (EPI)AFZ were, respectively, the numbers of (epi)catechin and (epi)afzelechin units contained in the proanthocyanidin molecule, and B were the numbers of B-type linkages between units.

### 2.5. In Vitro Antioxidant Activity

For the determination of the antioxidant activity, extracts (0.05 g) were treated with 10 mL of methanol/HCl (1000:1, *v*/*v*) by sonication for 5 min followed by an extra 15 min resting period. The mixture was then centrifuged (3024 g, 5 min, 5 °C) and filtered (0.45 µm). The radical scavenging activity of the extracts was determined by the ORAC (Oxygen Radical Absorbance Capacity) method using fluorescein as a fluorescence probe [13]. Briefly, the reaction was carried out at 37 °C in 75 mM phosphate buffer (pH 7.4) and the final assay mixture (200 µL) contained fluorescein (70 nM), 2,2'-azobis(2-methyl-propionamidine)-dihydrochloride (12 mM), and antioxidant (Trolox (1–8 µM) or phenolic extract (at different concentrations)). The plate was automatically shaken before the first reading and the fluorescence was recorded every minute for 98 min. A Polarstar Galaxy plate reader (BMG Labtechnologies GmbH, Offenburg, Germany) with 485-P excitation and 520-P emission filters was used. The equipment was controlled by the Fluostar Galaxy software version (v.4.11-0, BMG Labtechnologies GmbH, Offenburg, Germany) for fluorescence measurement. Black 96-well untreated microplates (Nunc, Denmark) were used. 2,2'-Azobis (2-methyl-propionamidine)-dihydrochloride and Trolox solutions were prepared daily and fluorescein was diluted from a stock solution (1.17 mM) in 75 mM phosphate buffer (pH 7.4). All reaction mixtures were prepared in duplicate and at least three independent runs were performed for each sample. Fluorescence measurements were normalized to the curve of the blank (no antioxidant). From the normalized curves, the area under the fluorescence decay curve (AUC) was calculated as:

$$\mathrm{AUC}=1+\sum_{i=1}^{i=98}\int {}_{i}/\int {}_{0}$$

where $\int {}_{0}$ is the initial fluorescence reading at 0 min and $\int {}_{i}$ is the fluorescence reading at time i. The net AUC corresponding to a sample was calculated as follows:

Net AUC = AUC_antioxidant_ − AUC_blank_

The regression equation between net AUC and antioxidant concentration was calculated. The ORAC value was estimated by dividing the slope of the latter equation by the slope of the Trolox line obtained for the same assay. Final ORAC values were expressed as mmol of Trolox equivalents (TE)/g of phenolic extract.

### 2.6. Cell Culture

The human gastric adenocarcinoma cell line AGS, human colorectal adenocarcinoma SW620 and monkey normal epithelial kidney cells Vero were obtained from American Type Culture Collection (ATCC, Rockville, MD, USA). They were grown in minimum essential Eagle’s medium (MEM) containing 10% fetal bovine serum (FBS) in the presence of 2 mmol/L glutamine, 100 IU/mL penicillin, 100 µg/L-streptomycin and 0.25 µg/mL amphotericin B. The cells were grown in a humidified atmosphere containing 5% CO_2_ at 37 °C and sub-cultured by detaching with trypsin–EDTA solution at about 70%–80% confluency. For the experiments, 100 µL of a cell suspension of 1.5 × 10^5^ cells/mL were seeded overnight into 96-well plates. The cells were further exposed for 48 h to various concentrations of *U. tomentosa* extracts (50 µL) in a humidified atmosphere containing 5% CO_2_ at 37 °C. For each experiment, the extract was dissolved in cell culture medium to a final concentration ranging between 15 and 500 µg/mL with a DMSO concentration below of 0.1 % (*v*/*v*). Control cultures were prepared with the addition of DMSO (vehicle control) to define the 100% of viability.

### 2.7. Assessment of Cytotoxicity by MTT Assay

After incubation for 48 h, MTT assays were performed to evaluate cytotoxicity. Briefly, the medium was eliminated, cells were washed once with 100 µL of PBS (Phosphate-Buffered Saline) and incubated with 100 µL of a MTT solution (3-(4,5-dimethylthiazolyl-2)-2,5-diphenyltetrazolium bromide; 0.5 mg/mL, final concentration) in PBS, for 2 h at 37 °C. Afterwards, MTT was removed, the formazan crystals formed were dissolved in 100 µL of ethanol 95%, and the absorbance was read at 570 nm in a microplate reader. For each extract concentration, the percentage of viable cells was calculated using the absorbance of the control (cells incubated with the vehicle solution (DMSO, 0.1 %)) as 100%. Dose-response curves were established for each extract and the concentration which is sufficient to reduce the cell number by 50% (IC_50_) was calculated. For each cell line, the extracts were tested in three independent experiments and in each experiment the different doses of extract were analysed in triplicate.

### 2.8. Antimicrobial Activity

Antimicrobial activity from phenolic extracts was evaluated on *Staphylococcus aureus* ATCC 25923, *Enterococcus faecalis* V583, and *Pseudomonas aeruginosa* PSP, which are bacteria characteristic of oral cavity and the respiratory system. *S. aureus* ATCC 25923 was obtained from the Spanish Type Culture Collection (CECT). *E. faecalis* V583 and *P. aeruginosa* PSP were clinical isolates obtained from human samples at the *Ramón y Cajal* Hospital (Madrid, Spain). Antibacterial assays were performed using a microdilution method in 96-well plates, according to Cueva et al. [14]. For each phenolic extract, a solution of 1mg/mL in sterilized water containing 10% DMSO (0.1 mg/mL final concentration on bacteria) was prepared. Eight serial dilutions were prepared so to get final concentrations of 100, 50, 25, 12.5, 6.25, 3.125, 1.562, 0.781 μg/mL. Inhibition percentages of the different extract concentrations were calculated by comparing the control growth with those obtained from cultures with phenolic extracts. Dose-response curves (% Inhibition versus extract concentration) were established for each extract and the concentration which was required to obtain 50% inhibition of growth (i.e., IC_50_ value) was calculated. For each bacteria strain, the extracts were tested in two independent experiments and in each experiment the different doses of extract were analysed in duplicate.

## 3. Results

### 3.1. Total Proanthocyanidin Content of U. tomentosa Extracts

As described in the methodology section, purification of *U. tomentosa* polar fractions with solvents of low and medium polarity allowed us to obtain phenolic enriched fractions of *U. tomentosa*. Total proanthocyanidin contents of the obtained phenolic extracts (*n* = 14) are summarized in Table 1. Findings indicated lower values for wood, ranging from 11.3 to 134.0 mg cyanidin chloride equivalents/g extract, and higher values ranging from 347.0 to 561.3 mg/g among all external parts (leaves, bark and stems), with values varying also according to plant origin. For instance, the highest values for the different plant parts (leaves, stems, bark and wood) clearly corresponding to extracts from the location of Sarapiqui whereas the lowest contents belonged to the Asomat location.

### 3.2. Proanthocyanidin Profile of U. tomentosa Extracts from Leaves, Stems, Bark and Wood

**Figure 1:**
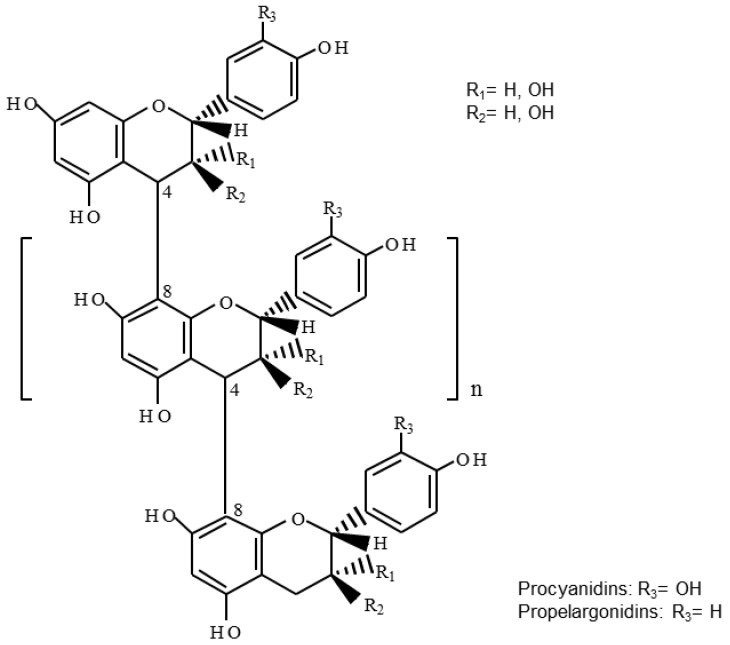
General chemical structure of B-type proanthocyanidins (composed by (epi)catechin units) and propelargonidins (composed by (epi)afzelechin units).

**Figure 2:**
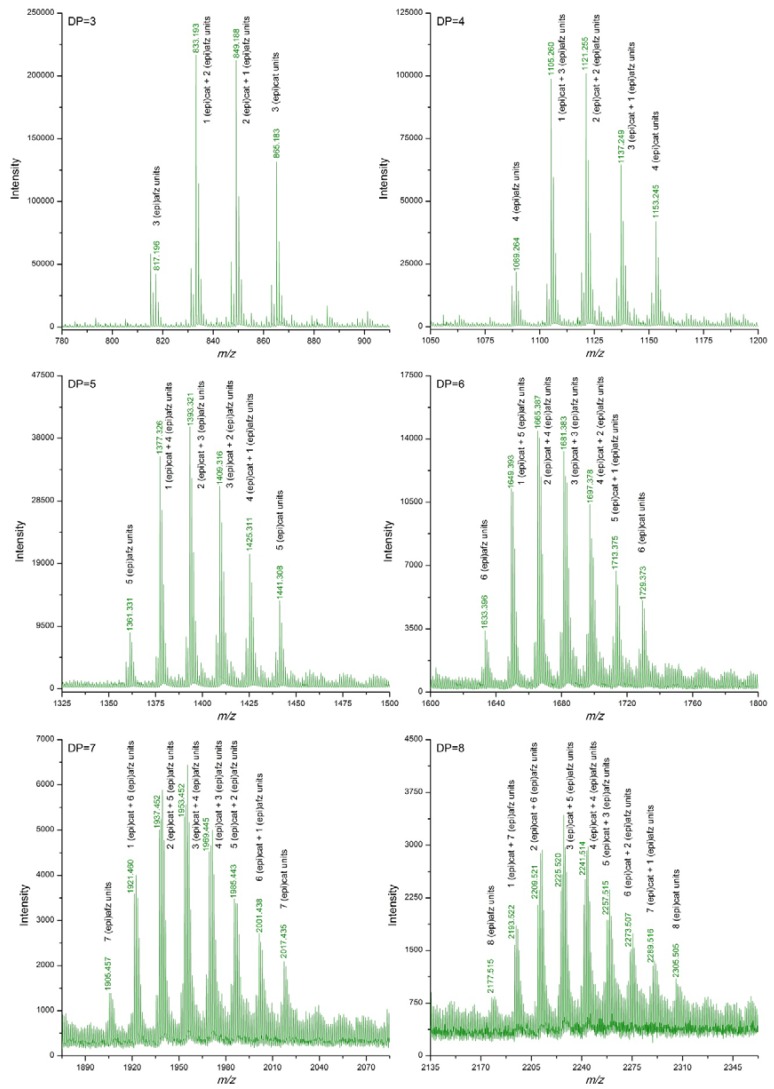
Enlargements of DI-ESI-QTOF mass spectrum of proanthocyanidins with DP 3–8 from a sample of *U. tomentosa* leaves from Sarapiquí location.

**Figure 3:**
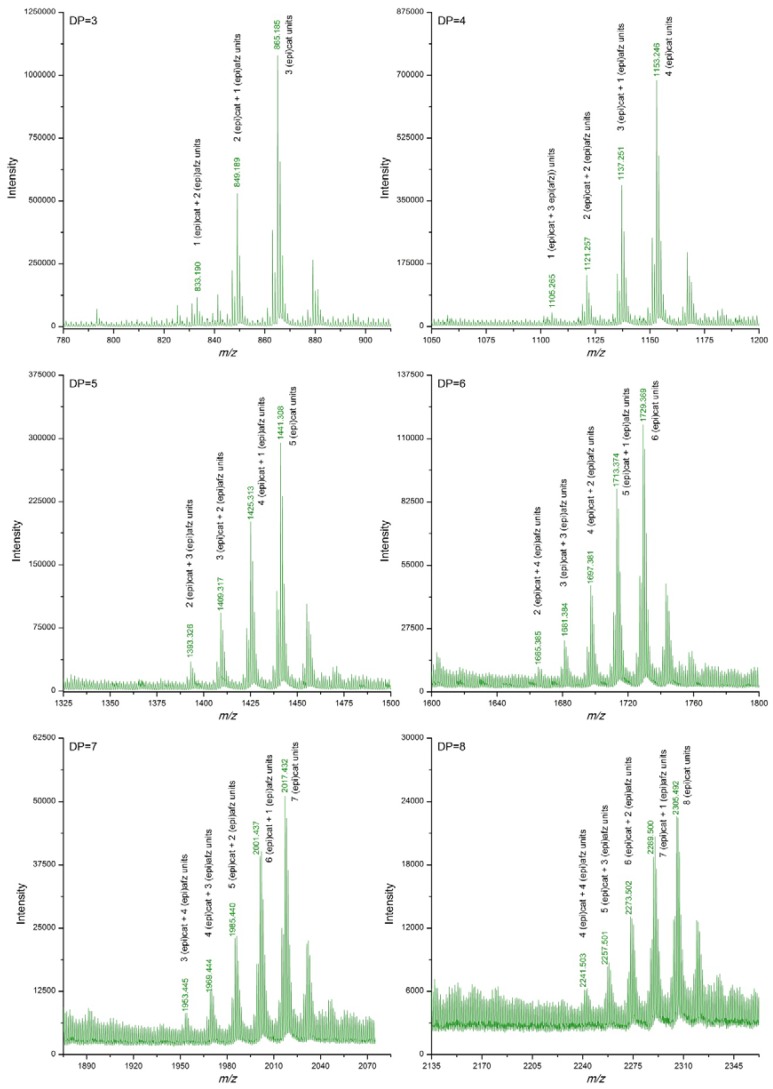
Enlargements of DI-ESI-QTOF mass spectrum of proanthocyanidins with DP 3–8 from a sample of *U. tomentosa* stems from Sarapiquí location.

**Figure 4:**
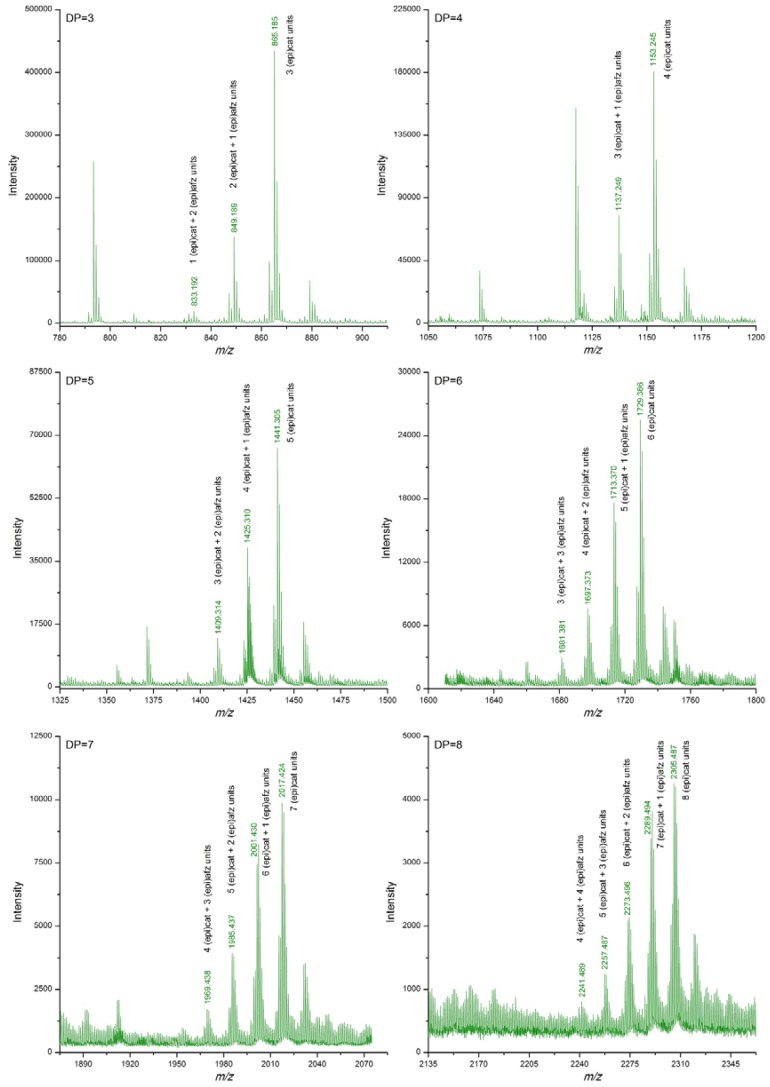
Enlargements of DI-ESI-QTOF mass spectrum of proanthocyanidins with DP 3–8 from a sample of *U. tomentosa* bark from Sarapiquí location.

Table 2 summarizes the *m/z* signals corresponding to [M−H]^−^ ions of proanthocyanidins detected in the *U. tomentosa* extracts following the DI-ESI-QTOF MS analysis described in the methodology. Theoretical *m/z* data are also included in Table 2. In addition, Figure 1, Figure 2, Figure 3 and Figure 4 show amplifications of mass spectra corresponding to leaves, stems, bark and wood extracts, respectively. For the four types of *U. tomentosa* extracts, proanthocyanidins resulted to be composed by units of (epi)catechin and/or (epi)afzelechin linked through B-type linkages (Figure 1). However, some differences in molecular composition and degree of polymerization (DP) were found among extracts from the different parts of the plant. Proanthocyanidins in extracts from *U. tomentosa* leaves included pure procyanidins -only composed by (epi)catechin units- with a DP between 3 and 9 units-, pure propelargonidins -only composed by (epi)afzelechin units- with a DP between 3 and 8 units, and mixtures of both of them -composed by (epi)catechin and (epi)afzelechin units-, with a DP up to 10 units (decamers) (Table 2, Figure 2). For instance, in the case of pentamers (DP = 5) six signals were detected, corresponding to pure procyanidins (5 units of (epi)catechin) (*m/z* = 1441.308), pure propelargonidins (5 units of (epi)afzelechin units) (*m/z* =1361,331), and mixed proanthocyanidins containing 4, 3, 2, or 1 units of (epi)catechin and, respectively, 1, 2, 3 or 4 units of (epi)afzelechin (*m/z* = 1425.311, 1409.316, 1393.321, 1377.326, respectively) (Table 2, Figure 2).

**Figure 5:**
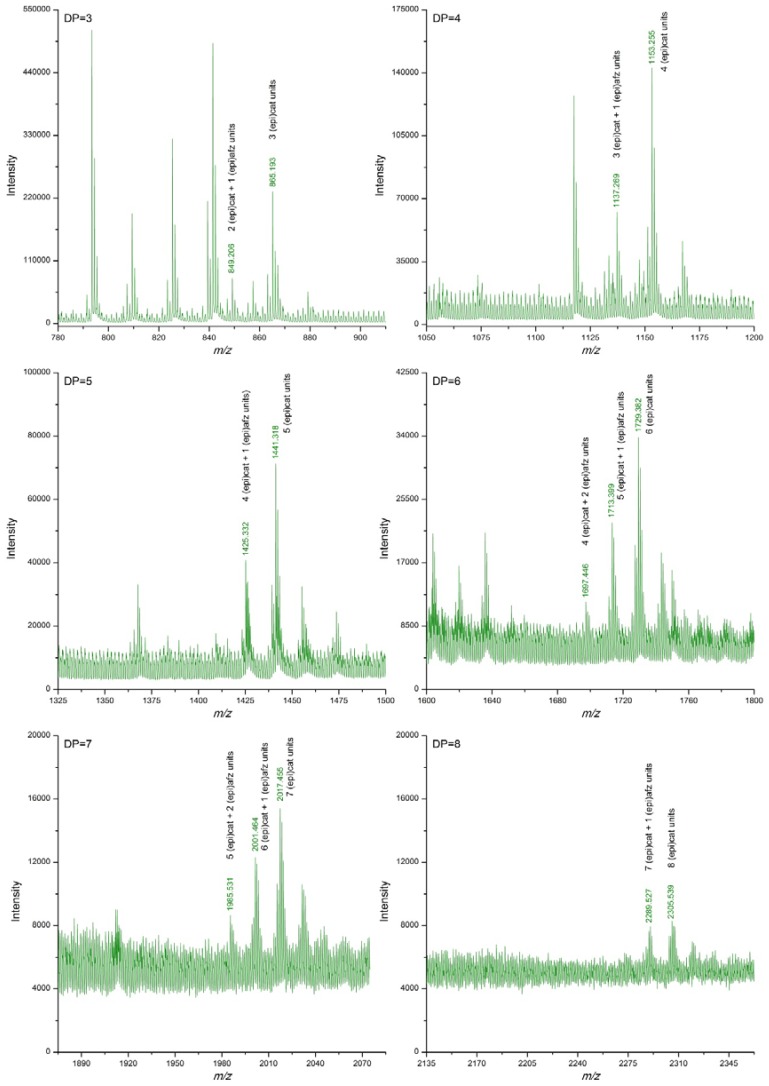
Enlargements of DI-ESI-QTOF mass spectrum of proanthocyanidins with DP 3–8 from a sample of *U. tomentosa* wood from Sarapiquí location.

Extracts from *U. tomentosa* stems contained proanthocyanidins up to decamers (DP = 10), either pure procyanidins or mixed proanthocyanidins with different (epi)catechin/(epi)afzelechin units ratio. In general, as the DP increased, signals corresponding to polymers with higher proportion of (epi)afzelechin units in relation to (epi)catechin units became non detected. No signals corresponding to pure propelargonidins were observed (Table 2, Figure 3). Similarly, extracts from *U. tomentosa* bark contained either pure procyanidins or mixed proanthocyanidins with different (epi)catechin/(epi)afzelechin units ratio, but both to at a DP of 11 units (Table 2, Figure 4). The proanthocyanidin profile of *U. tomentosa* wood extracts resulted simpler, being only detected signals corresponding to oligomers up to octamers, either consisted of pure procyanidins or mixed proanthocyanidins with 1 or 2 (epi)afzelechin units (Table 2, Figure 5). Whereas proanthocyanidin *m/z* signals followed a normal distribution for the different polymers detected in the leaves extracts (Figure 2), for stems, bark and wood extracts, higher signal intensities corresponded to pure procyanidins and proanthocyanidins with few units of (epi)afzelechin (Figure 3, Figure 4 and Figure 5).

### 3.3. Antioxidant Activity of U. tomentosa Extracts

**Figure 6:**
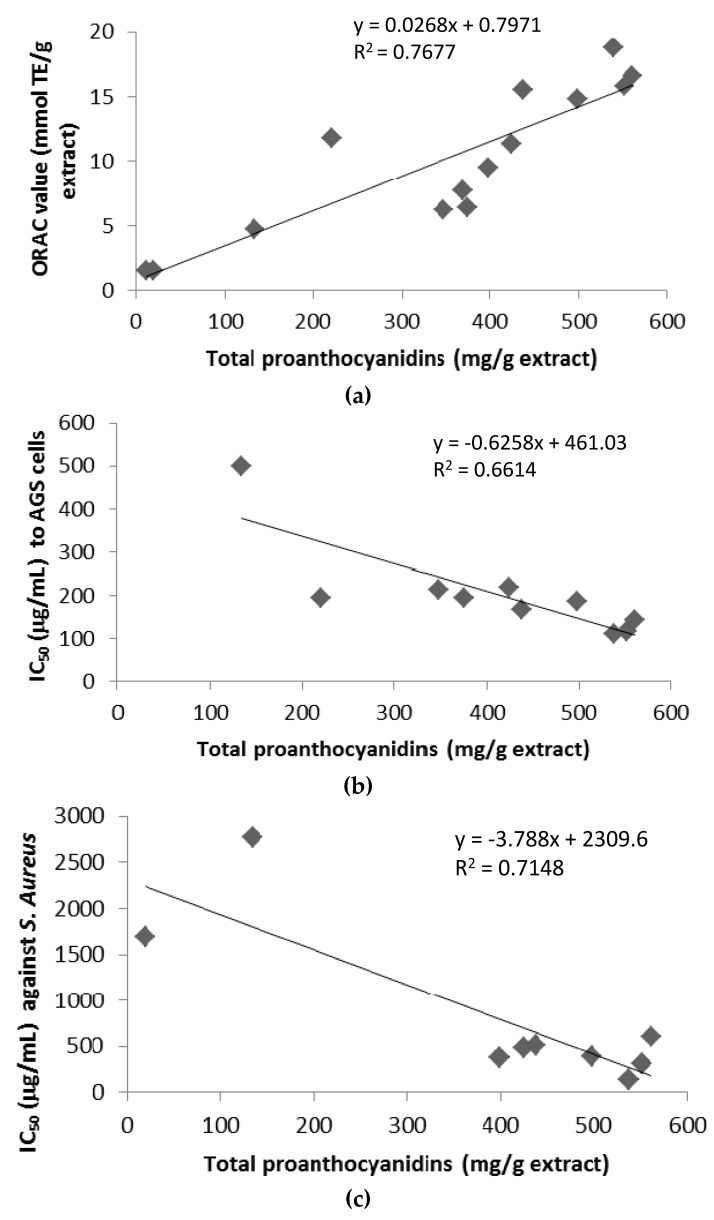
Linear correlation between total proanthocyanidin content and antioxidant capacity (ORAC value) (**a**), citotoxicity (IC_50_) to AGS cells (**b**), and antimicrobial activity (IC_50_) against *S. aureus* (**c**) of *U. tomentosa* extracts.

The antioxidant activity expressed as ORAC values of the *U. tomentosa* extracts varied in the order: leaves extracts (16.6–11.8 mmol Trolox equivalents/g) > stems (14.8–7.7 mol/mg) ~ bark (18.8–6.2 mmol/mg) > wood (4.7–1.5 mmol/mg) (Table 1). Among locations, Sarapiqui gave the highest values for the different type of extracts. In order to investigate if the proanthocyanidins contribute to the antioxidant activity of the extracts, a correlation analysis was carried out between the ORAC values and the total proanthocyanidin content (Table 1). A significant positive correlation was observed between them (*R*^2^ = 0.768) (Figure 6).

### 3.4. Citotoxicity of U. tomentosa Extracts

Table 3 reports the IC_50_ values for citotoxicity of the *U. tomentosa* extracts against the human gastric adenocarcinoma cell lines (AGS), human colon adenocarcinoma cell lines (SW620) and monkey normal epithelial kidney cells (Vero). IC_50_ values indicated that citotoxicity of *U. tomentosa* extracts resulted dependent on the cancer cell line used and also influenced by both the plant part and their collection site. Whereas growth of Vero cells were almost not affected by *U. tomentosa* extracts (IC_50_ > 500 µg/mL, with the only exception of leaves extracts from Los Chiles (IC_50_ = 458 µg/mL) and bark extracts from Sarapiquí (IC_50_ = 468 µg/mL)), growth of AGS and SW620 cells was strongly inhibited by all *U. tomentosa* extracts (IC_50_ < 220 µg/mL), except wood extracts that showed no cytotoxic effect towards any of the cell lines tested (Table 3). Among parts of the plants, IC_50_ variation intervals were quite similar for leaves and bark extracts: 116–195 and 111–220 µg/mL, respectively for leaves and bark extracts towards AGS cells, and 118–160 and 111–142 µg/mL respectively for leaves and bark extracts towards SW620 cells (Table 3). Of remarkable cytotoxicity for both adenocarcinoma cell lines were the extracts from leaves collected at Los Chiles (IC_50_ = 116 and 120 µg/mL respectively for AGS and SW620 cells) and from bark collected at Sarapiquí (IC_50_ = 111 and 142 µg/mL respectively for AGS and SW620 cells) (Table 3). As mentioned above, to investigate if proanthocyanidins contribute to the cytotoxicity of these extracts, a linear correlation analysis was carried out between the IC_50_ values and the total proanthocyanidin content (Table 1). A significant negative correlation was observed in the case of the AGS cells (*R*^2^ = 0.661) (Figure 6), but no significant correlation was found for the SW620 cells (data not shown).

### 3.5. Antimicrobial Activity of U. tomentosa Extracts

Antimicrobial activity of the *U. tomentosa* extracts was evaluated against three pathogenic strains known to be allocated in the oral cavity (*Staphylococcus aureus* ATCC 25923, *Enterococcus faecalis* V583 and *Pseudomonas aeruginosa* PSP) (Table 4). Most of the extracts were active against these different bacteria strains in the conditions used in this study. IC_50_ values varied between 133 and 6831 µg/mL and were influenced by both the part of the plants used and their location. Antimicrobial activity of extracts from Sarapiquí were in the order of bark > stems > leaves > wood for the three strains tested, whereas extracts from Palacios followed the order of stems > bark > leaves > wood for *S. aureus* ATCC 25923 and wood > leaves > stems > bark for *E. faecalis* V58 and *P. aeruginosa* PSP. In general, *S. aureus* ATCC 25923 and *P. aeruginosa* PSP were more sensitive (lower IC_50_ values) to the antimicrobial action of the *U. tomentosa* extracts than *E. faecalis* V583. As seen for the cytotoxic activity on AGS cells, significant negative correlations were observed between IC_50_ values and the total proanthocyanidin content for *S. aureus* ATCC 25923 (*R*^2^ = 0.715) (Figure 6). No significant correlations were found in the case of *E. faecalis* V583 either *P. aeruginosa* PSP (data not shown).

## 4. Discussion

Our previous studies on *U. tomentosa* [10,15] focused on the LC-ESI MS identification of low molecular weight polyphenols and demonstrated for the first time the occurrence of propelargonidin dimers in *U. tomentosa* extracts from different part plants. ^13^C-NMR studies also confirmed the presence of signals corresponding to epi(afzelechin) units in the different plant extracts. In the present study, we have carried out the characterization of oligomers and polymers of proanthocyanidins in *U. tomentosa* extracts from leaves, stem, bark and wood by accurate mass spectrometry (Q-TOF), and findings indicated that proanthocyanidins in *U. tomentosa* are composed of homopolymers of either (epi)catechin or (epi)afzlechins, as well as by heteropolymers constituted by both structural monomeric units. The occurrence of propelargonidins has been reported in food sources in lower quantities and dispersion than procyanidins, including A-type heteropolymers up to pentamers in strawberries [16] and up to undecamers with one (epi)afzelechin unit in cinnamon bark [17]; B-type propelargonidins in avocado fruit [18], kiwifruit pericarp [19] and raspberry [20], up to trimers in buckwheat grain [21] and boysenberry [22], up to tetramers in rhubarb [23], and up to heptamers with one (epi)afzelechin unit in almonds skins [24]. Some few reports exist in plants, including heteropolymers up to trimers in the leaves of *Fagus silvatica* [25] and *Senna alata* [26]. Studies in *Rumex acetosa* leaves showed predominance 5:1 of procyanidin over the propelargonidin units [27]; and while heteropolymers have been reported in *Delonix regia* leaves, only procyanidins existed in its bark [28]. In *Acacia confuse* and *Casuarina equisetifolia* bark, heteropolymers occurrence has been reported with predominance of procyanidins over propelargonidins [29,30]. Our results indicate a greater structural polydispersion in *U. tomentosa* proanthocyanidins, which was largely dependent on the part of plant, the complexity and DP range decreasing from the aerial parts of the plant to the inner wood. In our studies, the occurrence of homopolymers of properlagonidin up to DP 8 seems to be a very unique feature of *U. tomentosa* leaves as well as the presence of heteropolymers up to DP 10 with predominance 9:1 of (epi)afzelechin over the (epi)catechin units (Table 2). Besides the traditional use of *U. tomentosa* as an alkaloid source, our results demonstrated the potentiality of the plant as a source of proanthocyanidins, opening the possibilities of new applications in the nutraceutical industry [15], in particular for the leaves which has been neglected as source of bioactive components of *U. tomentosa* until now.

Previous studies indicated antioxidant activity of *U. tomentosa* aqueous and ethanol extracts with in-vitro DPPH, ABTS and TEAC tests [4,31,32] as well as of hydroethanol extracts under in vivo experiments [33]. These results appeared to be attributed to alkaloids, triterpenes and phenolic compounds, although these extracts were not fully characterized. Our study demonstrated for the first time the antioxidant activity of the proanthocyanidin enriched-fractions of *U. tomentosa*. ORAC values obtained for *U. tomentosa* leaves, stems and bark extracts (6.2–18.8 mmol Trolox/g extract) were similar to those of proanthocyanidin extracts from grape seeds [34], indicating once again the potential value of these extracts.

Considering that proanthocyanidins are known to reach the colon in intact form, anticancer cytotoxicity was evaluated in human gastric adenocarcinoma AGS and human colon adenocarcinoma SW620. In our case, results showed cell-dependence, since a negative correlation with PRO contents was observed for AGS gastric cells while no correlation was found for SW620 colon cells. *U. tomentosa* cytotoxic activity has been described mainly on alkaloids and triterpene extracts [2,35], however Pilarski et al. [36] studied triterpene-free and alkaloid-free *U. tomentosa* extracts on Lewis Lung carcinoma LL/2 cell lines, colon adenocarcinoma SW707, breast carcinoma MCF7, and cervical carcinoma KB, indicating that anticancer activity could be attributed to other secondary metabolites in *U. tomentosa* besides alkaloids. In addition, other reports indicate that fruits such as strawberry and raspberry containinig proanthocyanidins with (epi)catechin and (epi)afzelechin (propelargonidin) units have cytotoxicity activity on human colorectal adenocarcinoma HT-29 [37], for instance with antiproliferation EC_50 _values ranging from 110 to 120 mg/mL in the aforementioned strawberry and kiwifruit containing procyanidins and properlagonidins. Our results are in agreement demonstrating activity when using *U. tomentosa* extracts with propelargonidin and procyanidin oligomeric contents and showing high selectivity in both cancer cell lines in respect to normal vero cells. Regarding correlation of polyphenolic contents with cytotoxicity some studies argue in favour [38] while others [39] claim there is no correlation.

As stated earlier, *U. tomentosa* antimicrobial activity has received less attention and further, studies have reported divergent results. For instance, Ccahuana-Vasquez et al. [5] found that micropulverized *U. tomentosa* inhibited *Staphylococus* spp but failed to inhibit *P. aeruginosa* while another study [40] showed that *U. tomentosa* gel had an inhibitory effect on *Staphylococus* spp but was not active towards *E. faecalis*, suggesting that *U. tomentosa* type of extract may affect bacterial inhibition [2]. However, Herrera et al. [40] proved the antibacterial effect of *U. tomentosa* against *E. faecalis* in infected dentin. Our results align with these findings and evidence that antimicrobial activity varied according to *U. tomentosa* part, location and results suggested strain dependence. For instance, *S. aureus* was inhibited by all leaves, bark and stems extracts. Among them, Sarapiqui bark exhibited the strongest inhibition and was also the more effective extract towards *E. facealis* and *P. aeruginosa* strains. However, in overall, leaves -richer in proanthocyanidin contents with higher diversity- were the part that showed activity towards all three strains. These results align also with the ones of Cueva et al. [2] that showed proanthocyanidin oligomeric-rich extracts displaying stronger antimicrobial effects on both gram-positive and gram-negative strains. Therefore, these results suggest that proanthocyanidins would be, at least partly, responsible for these activities of the extracts, along with some other bioactive components such as low-molecular weight polyphenols, present in *U. tomentosa* extracts [10].

## 5. Conclusions

In conclusion, this paper constitutes the first detailed report of proanthocyanidin oligomers in *U. tomentosa* leaves, bark, stems and wood. Our findings indicate in general a rich and diverse procyanidin and propelargonidin oligomeric contents up to eleven monomeric units of (epi)catechin and (epi)afzelechin, in the extracts studied, as well as important antioxidant activity; cytotoxicity against gastric and colon cancer cell lines with selectivity in respect to vero normal cells; and antimicrobial effect against respiratory pathogens *S. aureus*, *E. faecalis* and *P. aeruginosa*. In addition, leaves demonstrated to be the part with widespread and better results in all activities studied, while individually leaves from Los Chiles and bark from Sarapiqui yield the best results. These findings suggest the potential value of *U. tomentosa* -especially leaves and bark- polyphenolic extracts with prospective use as functional ingredients.

## Figures and Tables

**Table 1:** Total proanthocyanidins and antioxidant activity of leaves, stems, bark and wood extracts from *U. tomentosa*.

Sample Location	Total Proanthocyanidins (mg/g Extract) ^1^	ORAC Value (mmol TE/g Extract) ^2^
**Leaves**		
Asomat	220.9 ± 0.5	11.8 ± 0.2
Los Chiles	551.5 ± 18.3	15.8 ± 0.2
Palacios	437.2 ± 36.9	15.5 ± 0.6
Sarapiquí	561.3 ± 13.5	16.6 ± 1.2
**Stems**		
Asomat	368.9 ± 3.3	7.7 ± 0.1
Palacios	398.7 ± 13.3	9.5 ± 0.3
Sarapiquí	497.8 ± 27.8	14.8 ± 0.1
**Bark**		
Asomat	347.0 ± 18.8	6.2 ± 0.1
Los Chiles	374.3 ± 12.1	6.4 ± 0.2
Palacios	424.4 ± 0.1	11.4 ± 1.1
Sarapiquí	538.0 ± 29.2	18.8 ± 0.2
**Wood**		
Asomat	11.3 ± 4.3	1.5 ± 0.0
Palacios	19.4 ± 2.2	1.5 ± 0.1
Sarapiquí	134.0 ± 4.8	4.7 ± 0.3

^1^ mg cyanidin chloride equivalents/g extract. ^2^ mmol Trolox equivalents/g extract.

**Table 2:** Proanthocyanidin characterization of *U. tomentosa* extracts by DI-ESI-QTOF MS.

DP	(epi)cat	(epi)afz	*m/z* Theoretical [M−H]^−^	*m/z* Experimental [M−H]^−^			
				Bark (*n* = 4)	Leaves (*n* = 4)	Stems (*n* = 3)	Wood (*n* = 3)
3	3	0	865.198	865.185	865.183	865.185	865.193
	2	1	849.203	849.189	849.188	849.189	849.203
	1	2	833.208	833.192	833.193	833.190	
	0	3	817.213		817.196		
4	4	0	1153.261	1153.245	1153.245	1153.246	1153.255
	3	1	1137.266	1137.249	1137.249	1137.251	1137.269
	2	2	1121.272		1121.255	1121.257	
	1	3	1105.277		1105.260	1105.265	
	0	4	1089.282		1089.264		
5	5	0	1441.325	1441.305	1441.308	1441.308	1441.318
	4	1	1425.330	1425.310	1425.311	1425.313	1425.332
	3	2	1409.335	1409.314	1409.316	1409.317	
	2	3	1393.340		1393.321	1393.326	
	1	4	1377.345		1377.326		
	0	5	1361.350		1361.331		
6	6	0	1729.388	1729.366	1729.373	1729.369	1729.382
	5	1	1713.393	1713.370	1713.375	1713.374	1713.399
	4	2	1697.398	1697.373	1697.378	1697.381	1697.446
	3	3	1681.403	1681.381	1681.383	1681.384	
	2	4	1665.409		1665.387	1665.385	
	1	5	1649.414		1649.393		
	0	6	1633.419		1633.396		
7	7	0	2017.452	2017.424	2017.435	2017.432	2017.455
	6	1	2001.457	2001.430	2001.438	2001.437	2001.464
	5	2	1985.462	1985.437	1985.443	1985.440	1985.513
	4	3	1969.467	1969.438	1969.445	1969.444	
	3	4	1953.472		1953.452	1953.445	
	2	5	1937.477		1937.452		
	1	6	1921.482		1921.460		
	0	7	1905.487		1905.457		
8	8	0	2305.515	2305.487	2305.505	2305.492	2305.539
	7	1	2289.520	2289.494	2289.516	2289.500	2289.527
	6	2	2273.525	2273.496	2273.507	2273.502	
	5	3	2257.530	2257.487	2257.515	2257.501	
	4	4	2241.535	2241.489	2241.514	2241.503	
	3	5	2225.540		2225.520		
	2	6	2209.545		2209.521		
	1	7	2193.551		2193.522		
	0	8	2177.556		2177.515		
9	9	0	2593.578	2593.547	2593.596	2593.563	
	8	1	2577.583	2577.547	2577.579	2577.571	
	7	2	2561.589	2561.553	2561.578	2561.568	
	6	3	2545.594	2545.560	2545.586	2545.563	
	5	4	2529.599		2529.582	2530.574	
	4	5	2513.604		2513.587		
	3	6	2497.609		2497.585		
	2	7	2481.614		2481.588		
	1	8	2465.619		2465.579		
10	10	0	2881.642	2881.618		2881.635	
	9	1	2865.647	2865.606		2865.632	
	8	2	2849.652	2849.642		2849.617	
	7	3	2833.657	2833.641	2833.696	2833.649	
	6	4	2817.662		2817.626		
	5	5	2801.667		2801.656		
	4	6	2785.672		2785.670		
	3	7	2769.677		2769.657		
	2	8	2753.682		2753.680		
	1	9	2737.687		2737.666		
11	11	0	3169.705	3169.693			
	10	1	3153.710	3153.685			
	9	2	3137.715	3136.719			

DP: Degree of polymerization; (epi)cat: (epi)catechin units; (epi)afz: (epi)afzelechin units; M: monoisotopic molecular mass.

**Table 3:** Cytotoxicity of *U. tomentosa* extracts to gastric (AGS) and colon (SW620) adenocarcinoma cells as well as to control Vero cells.

Sample Location	AGS Cells IC_50_ (μg/mL)	SW620 Cells IC_50_ (μg/mL)	Vero Cells IC_50_ (μg/mL)
**Leaves**			
Asomat	195 ± 14	118 ± 12	>500
Los Chiles	116 ± 5	120 ± 7	458 ± 17
Palacios	167 ± 17	160 ± 14	>500
Sarapiquí	145 ± 14	149 ± 29	>500
**Stems**			
Sarapiquí	188 ± 20	178 ± 13	>500
**Bark**			
Asomat	215 ± 23	111 ± 4	>500
Los Chiles	196 ± 17	111 ± 8	>500
Palacios	220 ± 13	132 ± 5	>500
Sarapiquí	111 ± 3	142 ± 5	468 ± 13
**Wood**			
Sarapiquí	>500	>500	>500

**Table 4:** Antimicrobial activity of *U. tomentosa* extracts against potential respiratory pathogens.

IC_50_ (μg/mL)			
Sample Location	*S. aureus* ATCC 5923	*E. faecalis* V583	*P. aeruginosa* PSP
**Leaves**			
Los Chiles	312	1164	1012
Palacios	516	1444	521
Sarapiquí	603	2095	475
**Stems**			
Palacios	380	6831	n.e
Sarapiquí	396	1383	417
**Bark**			
Palacios	490	n.e.	n.e.
Sarapiquí	133	395	152
**Wood**			
Palacios	1688	1112	449
Sarapiquí	2774	n.e.	811
